# Inside help for brain tumors: macrophage-mediated myelin recycling promotes cell state-specific glioblastoma progression

**DOI:** 10.1038/s41392-024-02055-0

**Published:** 2024-12-04

**Authors:** Antonio C. Pagano Zottola, Thomas Daubon, Varun Venkataramani

**Affiliations:** 1 University of Bordeaux, INSERM, U1312 BRIC, Pessac, France; 2 University of Bordeaux, CNRS, IBGC UMR 5095, Bordeaux, France; 3 Neurology Clinic and National Center for Tumor Diseases, University Hospital Heidelberg, Heidelberg, Germany

**Keywords:** Cancer microenvironment, CNS cancer

**Fig. 1:**
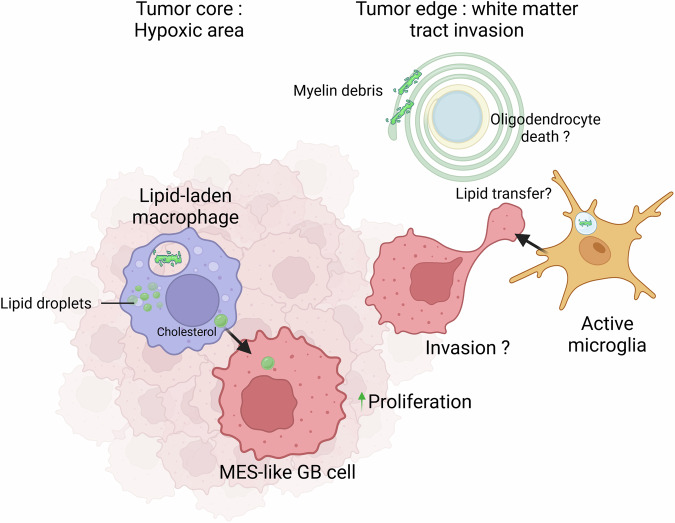
The role of crosstalk between macrophages, oligodendrocytes via myelin and glioblastoma cells promotes cell state-specific glioblastoma proliferation

In a recent paper published in *Cell*, Kloosterman et al. uncovered how lipid-laden macrophages in brain tumor called glioblastomas engage in myelin recycling, demonstrating that these cells process myelin debris into lipid resources that fuel the growth of mesenchymal-like glioblastoma cells while simultaneously maintaining an immunosuppressive microenvironment^1^ (Fig. 1). This finding suggests a promising therapeutic strategy, as pharmacological inhibition of lipid uptake through CD36 blockade improved survival in preclinical models and could potentially enhance the efficacy of existing treatments, including immunotherapy.

Glioblastoma (GB) is an aggressive, yet incurable brain tumor with a median survival of 8 months. Standard treatment typically involves surgical resection followed by radio-and chemotherapy. However, tumor recurrence is almost universally observed.^2^ Over the last decades, extensive research and numerous clinical trials have yielded only minor advances in treatment outcomes including immunotherapeutic approaches, with limited improvement in patient survival rates. GB has been shown to be characterized by molecular and cellular heterogeneity as well as an immunosuppressive microenvironment, predominantly shaped by tumor-associated macrophages and microglia.^2^

Kloosterman et al.^1^ uncovered myelin engulfment by macrophages and how this phenomenon promotes cell state-specific GB progression. By utilizing two genetically engineered mouse models, they identified eight distinct TAM subpopulations within the GB microenvironment. Among these, a notable increase was observed in metabolically active, lipid-laden macrophages (LLM) that were predominantly located within the hypoxic niches of GB associated with mesenchymal (MES)-like GB cells. LLMs exhibited an immunosuppressive phenotype, marked by altered chromatin landscapes and diminished expression of inflammatory genes.

Mechanistic investigation using ex vivo analyses into the generation of LLMs demonstrated that the lipid droplets needed are derived from myelin debris. Myelin, composed of 70–85% lipids (40% cholesterol, 40% phospholipids, and 20% glycolipids) and of proteins (15–30%), represents the largest reservoir of cholesterol and fatty acids within the brain. Myelin debris is scavenged by LLMs and myelin-derived lipids transferred to GB cells. The redistribution process led to increased proliferation of MES-like GB cells, which exhibit low cholesterol biosynthesis capacities and rely on external lipid sources. By processing the large quantities of lipids generated by demyelination, LLMs converted a potentially lipotoxic effect into a fuel source that sustains tumor growth. While lipids support GB cells in synthesizing cellular membranes, they simultaneously modulate epigenetic programs of LLMs, increasing levels of H3K27me3 and reinforcing their immunosuppressive phenotype by reducing chromatin accessibility. Moreover, myelin recycling within LLMs impaired cholesterol biosynthesis, resulting in the accumulation of cholesterol precursors. These precursors, through activation of Liver X receptors, induced the expression of lipid transporters such as ABCA1 and ABCG1, which were necessary for lipid redistribution in the tumor interstitial fluid.

Therapeutically, the study highlighted the potential of disrupting the lipid exchange between macrophages or microglia and cancer cells as a strategy to inhibit GB progression. Pharmacological inhibition of the lipid receptor CD36 after radiotherapy was shown to extend survival in GB-bearing mice by reducing the frequency of LLMs. The prognostic significance of the LLM signature was investigated by analyzing public databases, and higher LLM levels were associated with poorer outcomes in GB patients and predicted reduced responsiveness to immune checkpoint blockade therapy across various cancers.

These findings suggest that metabolic crosstalk between GB cells and LLMs exists and that targeting this reciprocal interaction could open new therapeutic avenues to combat GB and improve the efficacy of existing treatments, such as immunotherapy.

The presence of LLMs in the brain has so far been documented exclusively in the context of neurodegenerative diseases, where oligodendroglial dysfunction and subsequent demyelination have been observed.^3^ However, the influence of cancer cells and their microenvironment on the integrity of the myelin sheath, and whether this effect is localized to specific brain regions, remains unclear. It is plausible to hypothesize that this myelin degeneration results from ischemia, neoangiogenesis, and/or edema produced by the expanding tumor mass^4^ and its secretome, rich in glutamate and metalloproteinases—both toxic to oligodendrocyte lineage cells.^5^ Through a multi-omics approach, the authors have elucidated the spatial diversity of TAM subsets, depicting their role in the transition of GB cells from a less to more aggressive phenotype during therapeutic resistance, focusing on the hypoxic core of the GB. They further explored how myelin-loaded macrophages are able to shield tumor cells from toxic effects of high concentrations of myelin and further promote proliferation by exchanging lipids.

As discussed by the authors, myelin has a complex structure, and further investigation is needed to fully understand the role of additional myelin components, such as proteins, in cancer progression. Proposing LLMs as a therapeutic target in GB is intriguing. The approach aimed at targeting CD36, a lipid transporter overexpressed in LLMs, has shown promise, while the specificity of this approach to the inhibition of this complex crosstalk will needer further validation. Along this line, the identification of more LLMs-specific therapeutic targets could be a promising and innovative strategy for treating GB. Another important area of research is how other cancer cell states and brain tumor regions, such as the pathophysiologically relevant infiltrative edge, are affected by the described complex interplay of macrophages, myelin, and cancer cells. It is unclear whether such a phenomenon would also play a role in sparsely tumor-colonized brain regions and whether they would play a role in tumor localizations such as the gray matter. Lastly, it will be important to explore the clinical-translational relevance for GB patients and to develop assays apart from scRNA-sequencing to infer whether the same type of cross-talk is taking place in the setting of tissues.

Taken together, the authors demonstrated a novel mechanism of how the tumor microenvironment induces the remodeling of TAMs. Inhibition of pro-inflammatory properties, alongside enhanced phagocytosis of mature oligodendrocyte membranes, drives tumor progression. In the emerging field of cancer neuroscience that investigates the interaction between the nervous system and cancers, this work adds another layer of complexity. It highlights the tripartite communication between the innate immune system, oligodendrocytes, and tumor cells, where specific cancer cell states are able to reprogram immune responses for their own benefit and further emphasizes that deciphering the complex multicellular networks in GB is an important frontier in brain tumor research.
